# An Exploratory Investigation of the Role of Openness in Relationship Quality among Emerging Adult Chinese Couples

**DOI:** 10.3389/fpsyg.2017.00382

**Published:** 2017-03-15

**Authors:** Yixin Zhou, Kexin Wang, Shuang Chen, Jianxin Zhang, Mingjie Zhou

**Affiliations:** ^1^Key Laboratory of Mental Health, Institute of Psychology, Chinese Academy of SciencesBeijing, China; ^2^School of Journalism and Communication, Tsinghua UniversityBeijing, China; ^3^Youth Research Institute, China Youth University of Political StudiesBeijing, China

**Keywords:** openness, intimate relationships, relationship quality, personality consistency, cross-level polynomial regression

## Abstract

This study tested emerging adult couples’ openness and its fit effect on their romantic relationship quality using quadratic polynomial regression and response surface analysis. Participants were 260 emerging adult dyads. Both dyads’ openness and relationship quality were measured. The result showed that (1) female and male openness contribute differently to relationship quality; (2) couples with similar high openness could experience better relationship quality than those with similar low openness traits; and (3) when dyadic openness is dissimilar, it is better to be either relatively high or relatively low than to be moderate. These findings highlight the role of openness in emerging adults’ romantic relationships from a dyadic angle.

## Introduction

Emerging adulthood is proposed to be a new conception of development for the period from the late teens through the twenties, with a focus on ages 18–25 (Arnett, 2000), when most people begin to move toward making the commitments that structure adult life: marriage, parenthood, and a long-term job (Arnett, 2004). At this life stage, young adults start learning how to attain and maintain a close relationship for the first time and that fostering close relationships plays as the most indispensable and profound role in building physical well-being as well as psychological health (Berscheid, 1999). One of the striking differences between young adolescents and emerging adults is in the place of romantic relationships in their lives (Montgomery, 2005). Personality is a construct that is vital to the understanding of relationship experiences (Hines and Saudino, 2008). Interpersonal situations, particularly close relationships, can be considered the central context in which personality expresses itself in our daily lives (Robins et al., 2002). In the present study, we aim to explore the relationship between openness fit and relationship quality among dating emerging adults. Our study has three contributions in this field. First, we focus on Chinese emerging adults since emerging adulthood varies by culture and in developing countries; for example, in China, emerging adulthood may be experienced more often in urban areas than in rural areas (Arnett, 2014). Meanwhile, although previous studies on the relationship between personality and relationship quality have used both individual samples and dyadic samples among marriage cohorts (Holland and Roisman, 2008; Dyrenforth et al., 2010; Mund and Neyer, 2014), the changing life stages, demands, and expectations outside the intimate relationship may lead to different effects on personality (Shiota and Levenson, 2007), so it is necessary to dedicate the research on the effect of personality on emerging adults’ romantic relationships. Thus, this study provided new knowledge on the emerging adults of an emerging country. Secondly, numerous studies have explored the effect of personality on determining relationship quality and found consistent crucial effects of neuroticism and some protective effects of conscientiousness, agreeableness, and extraversion (Furler et al., 2014; Mund and Neyer, 2014; Schaffhuser et al., 2014a). In this study, we focused on the openness personality trait to predict the dating emerging adults’ relationship quality. Although openness also has numerous influences on social and interpersonal phenomena (McCrae, 1996), we still know little about the effect of openness on the course of intimate and personal relationships (McCrae and Costa, 1997). Thirdly, previous studies highlight the mutual influences between couples (Barelds, 2005; Kenny and Ledermann, 2010) as well as their similarities in personality (Gonzaga et al., 2007; Shiota and Levenson, 2007; Cuperman and Ickes, 2009; Arránz Becker, 2013). Personality similarity research often relies on methods that collapse two personality measures into a single score intended to represent similarity. However, these methods suffer from numerous methodological problems using a single score to predict the outcome, which would cover up and limit the prediction of each dyad’s personality (Edwards, 2001). For example, the female openness score that is higher than the male score may reach the same similarity score because male openness is higher than female openness within each dyad. Therefore, when we focused on the openness fit, compared to just considering the similarity or dissimilarity, we accounted for various situations of fit in this study.

### The Interpersonal Aspect of Openness

Openness is usually portrayed as an intrapsychic dimension that describes individual differences in the structure and function of the mind (McCrae, 1996). It is considered a reflection of creativity, artistic interest, emotionality, adventurousness, intellect, and liberal thinking (Costa and McCrae, 1992; Baer et al., 2008). Openness can further be divided into openness to creative intellect experiences and psychological openness in the motivational, social, and emotional systems (McCrae and Sutin, 2009; Woo et al., 2014). In the conceptualization of openness, intrapsychic aspects such as the intellect and perception have been primarily emphasized (Deyoung et al., 2012; Deyoung, 2015). It is better understood as an essential way of approaching the world that affects internal experience, accompanied by interpersonal interactions and social behavior (McCrae, 1996). That is to say, in addition to the intrapsychic aspects, a consideration of openness in all social fields illustrates a second, interpersonal aspect of openness. A meta-analytic review also sheds light on the interpersonal domain of openness and illustrated a positive association between openness and interpersonal sensitivity (Hall et al., 2009). However, the interpersonal aspect of openness has not been well documented (Ozer and Benet-Martinez, 2006).

### Openness and Relationship Quality: Individual Approach

Past research on the association between individual characteristics and close relationship quality has taken either an individual or a dyadic approach (Luo et al., 2008). The individual approach aims to examine the link between self and/or partner characteristics (e.g., personality) and relationship quality. Substantial research finds weak or even non-correlations between one’s openness trait and self-evaluated relationship quality in both the cross-sectional and longitudinal sample (Karney and Bradbury, 1995b; Watson et al., 2000; Glicksohn and Golan, 2001; Noftle and Shaver, 2006; Furler et al., 2014). However, Solomon and Jackson (2014) explored that a higher level of openness could not only predict a lower marriage satisfaction but also a higher possibility of dissolution 4 years later. Still other studies based on dating couples illustrated that openness was one of the two most valued personality characteristics by both sexes in the mate-selecting process (Botwin et al., 1997). As for the partner characteristics, the previous results were inconsistent. For example, Holland and Roisman (2008) found that the partner’s openness could positively predict relationship satisfaction, while Schaffhuser et al. (2014a) demonstrated that the partner’s openness trait was a negative predictor of relationship quality. Furthermore, the partner effect might be asymmetrical to the gender. According to Neyer and Voigt (2004) findings, the male’s openness could benefit his female partner’s perceived relationship quality, but not vice versa. Two other studies showed that individuals who have a mate with a high level of openness are more satisfied with their marriage in newlywed couples (Botwin et al., 1997; Watson et al., 2004). Thus, the effects of openness on the quality of a relationship are still unclear.

### Openness and Relationship Quality: Dyadic Approach

Although findings from the individual approach are important for our understanding of the effect of personality on relationship quality, this approach treats two dyads’ personalities as two independent variables without taking the “couple” into consideration. On the contrary, the dyadic approach overcomes this limitation by shifting the focus from the individual to the couple by specifically addressing how one’s relationship quality may depend on dyadic characteristics (e.g., personality similarity) (Luo et al., 2008). There were two competitive hypotheses in the association between personality similarity and relationship quality: the similarity-attraction hypothesis and the complementary hypothesis. The similarity-attraction hypothesis regards the fact that communicating with a similar individual will raise people’s affection and decrease the barriers of interaction (Clore and Byrne, 1974; Berger and Calabrese, 1975). The complementary hypothesis suggests that individuals who prefer to seek partners with dissimilar characteristics to achieve novelty and complement will have more communication and individual growth (Baxter and West, 2003). In the aspect of openness, it is attested that the similarity in openness was associated with greater satisfaction in newlyweds (Watson et al., 2004) and married couples (Luo and Klohnen, 2005), and a profile-based openness similarity displayed both significant linear and quadratic relationships with wives’ marital satisfaction. On the contrary, in the study by Watson et al. (2000), Glicksohn and Golan (2001), and Barelds (2005), neither the openness similarity nor its satisfaction prediction was distinctly found in a sample consisting of both dating and married couples, and similar results were seen through middle- and old-aged groups (Shiota and Levenson, 2007). In summary, it is still unclear how openness and its consistency interact with relationship quality, leading researchers to conclude it as the only characteristic where no well-documented impact is observed in the interpersonal realm (Ozer and Benet-Martinez, 2006).

### Personality Fit Beyond Just (Dis)similarity

The relation between one dyad member’s self-reported personality and the self-reports of the other dyad member is generally described simply as a similarity (Lee et al., 2009). Personality similarity research often relies on methods that collapse two personality measures into a single score intended to represent similarity [i.e., absolute difference scores (ADS) or interaction terms based on two dyad scores; Luo and Klohnen, 2005]. However, both of these methods have been criticized. Using a single score to predict the outcome would cover up and limit the prediction of each dyad’s personality (Edwards, 2001). In most cases, difference scores are used to represent congruence (i.e., fit, match, similarity, or agreement; Edwards and Parry, 1993). For example, when we use the ADS (Turban and Jones, 1988; Morry, 2007) as the similarity index, we cannot distinguish certain situations because the similar high openness dating dyad and similar low openness dating dyad may receive the same scores on similarity indices; on the other hand, the female openness score that is higher than the male score may reach the same similarity score because the male openness is higher than the female openness within each dyad. What is important is that these similarity patterns may manifest differently in close relationships and cause different consequences, but hardly can they be described by simple difference scores. As to the interaction term of both dyads’ personalities, however, although a significant interaction does indicate that the effect of one dyad’s openness depends on the level of the other dyad’s openness, it does not necessarily indicate the presence of a similarity effect (Luo and Klohnen, 2005). Thus, as Griffin et al. (1999, p. 517) suggested, “Relationship researchers should tell the whole story and, like a successful movie director, try to squeeze all the information out of their source.”

### The Components of Relationship Quality

Several constructs were commonly used to refer to relationship quality, such as satisfaction, commitment, love, intimacy, trust, and passion. A previous study involving Chinese participants showed that Chinese people scored lower in passion than Westerners, while they did not differ in terms of commitment and intimacy (Gao, 2001). For the Chinese romantic relationships, the relationship satisfaction was significantly affected by intimacy and commitment, but not by passion (Ng and Cheng, 2010). Therefore, not surprisingly, dating relationships in Chinese societies seem to involve less hedonic aspiration but a larger degree of relational obligations and mutual respect than those in Western societies (Chan et al., 2012). Meanwhile, personality had diverse effects on components of relationship quality. A previous study on openness and relationship quality showed that openness had a positive effect on males’ intimacy and passion, but had little relation to males’ commitment and females’ relationship outcomes (Campbell and Kaufman, 2015). Therefore, it is necessary to distinguish the effect of openness on different components of relationship quality.

### The Hypotheses of Current Study

To sum up, as an exploratory study, this research aims to clarify openness and its fit effect on several components of couple-level relationship quality among Chinese emerging adults by considering individual and dyadic characteristics simultaneously. We hypothesize at both the individual and dyadic level.

#### At Individual Level

Although substantial studies showed weak or even non-correlations between openness and relationship quality regardless of cross-sectional or longitudinal sampling (Karney and Bradbury, 1995b; Watson et al., 2000; Noftle and Shaver, 2006), in this study, we focus on emerging adults’ (typically college students; Arnett, 2014) romantic relationships. The college environment exposes individuals to a diverse set of ideas, people, and cultural traditions, as well as sparks their curiosity and stimulates them to consider a wider range of perspectives and values (Robins et al., 2001; Furler et al., 2014). Hence, a college student may benefit from his or her high openness trait in various aspects. Furthermore, there were some research results that indicated that both sexes prefer to choose a mate who scores higher on openness among both dating couples and newlywed couples (Botwin et al., 1997), and openness could benefit the marriage satisfaction of both spouses among newlywed couples (Botwin et al., 1997; Watson et al., 2004). Therefore, we speculate that, in terms of the dating samples, it could be expected that individual-level openness could enhance relationship quality. We thus hypothesize that:

H1: The higher the openness in both males and females, the higher the relationship quality facets the couple would perceive.

#### At Dyadic Level

Although there are both the similarity-attraction hypothesis and complementary hypothesis explaining relationship quality, when it comes to the openness to experience, there is little evidence of a complementary hypothesis, whereas a number of studies have shown that similarity in openness was associated with greater satisfaction in newlyweds (Watson et al., 2004) and married couples (Luo and Klohnen, 2005). Hence, we assumed that:

H2: Compared to the incongruence, dyad congruence in openness would lead to a better quality in relationship facets.

When two dyads are congruent in openness, it is necessary to distinguish between two situations: both dyads with a high score in openness and both dyads with a low score in openness. Combined with *H1*, the inference is that dyads with a high score in openness would have better relationship quality than both dyads with a low score in openness. We then assumed that:

H3: Compared with both dyads with a high score in openness, both dyads with a low score in openness would suffer from a poorer quality in relationship facets.

At the same time, when two dyads are incongruous in openness, it is also necessary to distinguish between two opposite situations. The female openness score is higher than the male and the male openness score is higher than the female within each dyad. Since previous findings suggested that there might be a positive relationship between openness and average-level relationship quality (Barelds, 2005; Neyer and Voigt, 2004; Watson et al., 2004), it is expected that the high openness score of each dyad will promote relationship quality. We therefore assume that whether the male score is higher than the female score or the female score is higher than the male score, one dyad with relatively higher openness will compensate for the lack of openness in the other dyad in shaping the high relationship quality. Hence, we assume that:

H4: Those dyads with at least one side with a high openness score will achieve a better quality in relationship facets than those dyads whose openness traits are both at a moderate level.

## Materials and Methods

### Participants

As typical emerging adults are receiving higher education (Arnett, 2014), students from six universities in China were investigated. Before questionnaires were distributed, it was ensured that individuals were currently in a dating relationship and that their partners were also willing to participate. The final sample consisted of 260 heterosexual dating couples; the average age was 21 for men (*SD* = 2.24) and 21 for women (*SD* = 1.88). Under the instruction of the investigator, they answered the questionnaire independently at the same time.

### Measures

#### Personality

Young adults’ openness trait was measured by the 4-item Imagination/Intellect scale from Mini-IPIP (Donnellan et al., 2006): “Have a vivid imagination”; “Am not interested in abstract ideas (R)”; “Have difficulty understanding abstract ideas (R)”; and “Do not have a good imagination (R),” which were rated on a 5-point Likert-type scale, thus holding a high similarity with IPIP-FFM in predicting the openness traits (Donnellan et al., 2006). The Chinese version held a good psychometric qualities in the Chinese sample (Li et al., 2012). The Cronbach’s alpha is 0.67 in this study.

#### Relationship Quality

Fletcher et al.’s (2000) Perceived Relationship Quality Component (PRQC) was used to assess six aspects of relationship quality (relationship satisfaction, love, commitment, trust, intimacy, and passion) and included 18 items. The answers were recorded on a scale from 1 (*not at all*) to 7 (*extremely*). The Cronbach’s alpha for the total PRQC is 0.94, 0.85 for relationship satisfaction, 0.65 for love, 0.80 for commitment, 0.73 for trust, 0.81 for intimacy, and 0.75 for passion. Perceived relationship quality per couple was averaged by the data from both sides of the dyad. We used the average score as the indicator of the couple’s relationship quality for the following two reasons. First, the model of our research was couple-centered, not individual-centered, so the general scope of the couple’s relationship quality rather than the actor-partner perception was emphasized. Second, there is high interdependence between the male and female relationship quality scores (*r*_satisfaction_ = 0.54; *r*_love_ = 0.32; *r*_commitment_ = 0.41; *r*_trust_ = 0.47; *r*_intimacy_ = 0.57; *r*_passion_ = 0.48). Thus, the average score is a good representation of each couple’s relationship quality (Gonzaga et al., 2007).

#### Control Variables

Age, love status, and time spent together were controlled according to prior research. Couples living together may differ in dynamics, expectations, and homogeneity compared to couples in a long-distance relationship (Karney and Bradbury, 1995a). Participants were asked whether they were in a long-distance relationship (1 = “Long-distance relationship”; 2 = “Close-distance relationship”). In our sample, 22.7% were in long-distance relationships and 77.3% were in geographically proximal relationships. Also, we controlled the time spent together per week because it was found that investment in spousal interaction also affects romantic relationship quality and openness similarity (Watson et al., 2004; Masarik et al., 2013; Rauer et al., 2013). Time spent together per week was measured by a 7-point scale from “5 h and below,” to “5–10 h,” “10–20 h,” “20–30 h,” “30–40 h,” “40–50 h,” and “50 h and above,” and it was averaged by both members of the couple. Mean time spent together per week per couple was near 20–30 h (*M* = 3.76, *SD* = 1.84, see **Table 1**).

**Table 1:** Correlation analyses among length of relationship, time spent together, openness from both partners, and their relationship quality.

	*M*	*SD*	1	2	3	4	5	6	7	8	9	10	11	12
(1) Geographically proximal relationship	1.77	0.42	1											
(2) Time spent together	3.76	1.84	0.28ˆ***	1										
(3) Male age	21.40	2.24	0.10	0.15ˆ*	1									
(4) Female age	20.72	1.88	0.11	0.18ˆ**	0.76ˆ**	1								
(5) Male openness	3.40	0.66	0.02	0.17ˆ**	-0.04	0.01	1							
(6) Female openness	3.43	0.70	-0.07	0.08	-0.02	0.02	0.09	1						
(7) Satisfaction	5.65	0.96	0.17ˆ**	0.14ˆ*	0.06	0.11	0.10	0.09	1					
(8) Love	5.62	0.94	0.16ˆ**	0.16ˆ**	0.07	0.09	0.17ˆ**	0.05	0.77ˆ***	1				
(9) Commitment	5.74	0.86	0.18ˆ**	0.15ˆ*	0.10	0.15ˆ*	0.13ˆ*	0.07	0.85ˆ***	0.79ˆ***	1			
(10) Trust	5.67	0.87	0.20ˆ**	0.15ˆ*	0.11	0.13ˆ*	0.08	0.11ˆ*	0.78ˆ***	0.74ˆ***	0.82ˆ***	1		
(11) Intimacy	5.51	0.93	0.27ˆ***	0.21ˆ**	0.14ˆ*	0.19ˆ*	0.14ˆ*	0.12	0.80ˆ***	0.73ˆ***	0.81ˆ***	0.78ˆ***	1	
(12) Passion	4.89	0.96	0.20ˆ**	0.20ˆ**	0.17ˆ**	0.18ˆ**	0.09	0.12	0.63ˆ***	0.63ˆ***	0.63ˆ***	0.65ˆ***	0.80ˆ***	1


^∗^*p < 0.05, ^∗∗^*p* < 0.01, ^∗∗∗^*p* < 0.001*.

### Analytic Strategies

To explore the similarity effect of each dyad’s personality, most previous research has computed ADS or interaction terms based on two dyad scores. However, both of these methods have their limits. As to the ADS, for example, if Tom rates himself as moderately open (a score of 3) and his partner Rose rates herself as very open (a score of 5), their ADS would be 2, but if Tom rates himself as moderately open (a score of 3) and his partner Rose rates herself as very open (a score of 5), their ADS would be also 2. Meanwhile, if Tom rates himself as highly open (a score of 5) and his partner Rose also rates herself with a very high score on openness (a score of 5), their ADS would be 0, but if Tom rates himself with a low openness score (a score of 1) and his partner Rose also rates herself with a low openness score (a score of 1), their ADS would also be 0. In both situations, we could not distinguish them from ADS. The other method by which couple similarity has been assessed is by entering the interaction term after the couple’s individual scores have been entered. However, although a significant interaction does indicate that the effect of one spouse’s self-ratings depends on the level of the other spouse’s ratings, it does not necessarily indicate the presence of a similarity effect (Luo and Klohnen, 2005). Conversely, the polynomial regression model incorporates both forms of fit portrayed by the ADS and interaction term, allowing the data to determine the specific form of fit (Meilich, 2006). Thus, one should take advantage of polynomial regression (Edwards, 2002), which contains separate measures of both entities (e.g., female personality, male personality), supplemented by higher order terms (e.g., the squares of both side’s personality measures and their product), and then allows for more fine-tuned interpretations, which permits direct tests of commensurate predictors and illustrates the interaction pattern. This could answer how agreement, discrepancy, and direction of discrepancy influence the outcome (Shanock et al., 2010), thus taking both dyads’ openness and their similarity into account at the same time as predicting the relationship quality.

Following Edwards (2002) suggestion, we first conducted polynomial regression with SPSS 19.0. The control variable, linear and quadratic personality, and the interaction item of one couple (Male Openness × Female Openness) were put into hierarchical regression in three steps.

The increment in *R*^2^ was tested. If the increment in *R*^2^ from the quadratic and product terms was significant, then additional tests were conducted to subdivide the joint effects by testing the slope and curvature of the congruence (incongruence) line. This was calculated using the Excel spreadsheet from Shanock et al. (2010). Last, the underlying three-dimensional relationship between couples’ paired openness and relationship quality (Z) was depicted based on six coefficients: the linear and quadratic personality for both males and females, the interaction item of one couple (Male openness × Female openness), and the intercept of the polynomial regression using Matlab. The estimated polynomial regression was as follows:

*Z* = β_0_ + β_1_ × Male openness + β_2_ × Female Openness + β_3_ × Male openness^2^ + β_4_ × Male openness × Female openness + β_5_ × Female Openness^2^ + *e*.

Details about this method are provided elsewhere (Shanock et al., 2010; Zhang et al., 2012).

## Results

The correlation analysis between personality traits and relationship quality was conducted first (see **Table 1**). Analyses revealed that there were small effects between emerging adults’ openness and their relationship quality (the *r* range from 0.05 to 0.17).

**Table 2** demonstrates the polynomial regression of relationship quality on openness personality. In Model 1 (M1), we controlled geographically proximal relationship, time spent together every week, and age of the individuals, respectively. Analysis showed that geographically proximal relationship was distinguished in predicting relationship quality.

**Table 2:** Cross-level polynomial regression of relationship quality on openness congruence/incongruence^a^.

	Satisfaction			Love			Commitment			Trust			Intimacy			Passion		
Variables	M1	M2	M3	M1	M2	M3	M1	M2	M3	M1	M2	M3	M1	M2	M3	M1	M2	M3
Constant	4.27ˆ***	3.43ˆ***	3.16ˆ***	4.24ˆ***	3.23ˆ***	2.97ˆ***	3.96ˆ***	3.14ˆ***	2.87ˆ***	4.02ˆ***	3.23ˆ***	3.06ˆ***	2.99ˆ***	1.88ˆ*	1.66ˆ*	2.44ˆ***	1.53	1.29
Geographically proximal relationship	0.31ˆ*	0.34ˆ*	0.38ˆ**	0.28	0.33ˆ*	0.38ˆ*	0.28ˆ*	0.32ˆ*	0.36ˆ**	0.33ˆ*	0.35ˆ**	0.37ˆ**	0.49ˆ***	0.53ˆ***	0.57ˆ***	0.34ˆ*	0.37ˆ*	0.41ˆ**
Time spent together	0.05	0.03	0.03	0.06	0.04	0.04	0.06	0.06	0.06	0.04	0.03	0.02	0.06	0.04	0.04	0.07ˆ*	0.06	0.06
Male age	-0.02	-0.01	-0.01	-0.01	0.00	0.01	0.05	0.03	0.02	0.01	0.01	0.01	-0.01	0.00	0.01	0.03	0.03	0.04
Female age	0.05	0.05	0.04	0.04	0.03	0.02	-0.01	-0.01	0.00	0.04	0.03	0.03	0.08	0.07	0.07	0.05	0.04	0.04
Male openness		0.14	0.18		0.24ˆ**	0.28ˆ**		0.17ˆ*	0.22ˆ**		0.10	0.15		0.19ˆ*	0.22ˆ*		0.12	0.15
Female openness		0.10	0.10		0.04	0.03		0.06	0.06		0.12	0.12		0.13	0.12		0.14	0.14
Male openness^2^			-0.10ˆ*			-0.06			-0.09ˆ*			-0.06			-0.06			-0.06
Male openness × Female openness			-0.06			-0.10			-0.09			-0.15ˆ**			-0.08			-0.04
Female. openness^2^			0.10ˆ*			0.15ˆ**			0.11ˆ*			0.09			0.11ˆ*			0.11ˆ*
*R*^2^	0.05	0.06	0.10	0.04	0.07	0.12	0.06	0.08	0.13	0.06	0.07	0.13	0.11	0.14	0.18	0.08	0.10	0.13
Δ*R*^2^		0.01	0.04ˆ*		0.03ˆ*	0.05ˆ**		0.02	0.05ˆ**		0.01	0.06ˆ**		0.02ˆ*	0.04ˆ*		0.02	0.03
Congruence (M = F) line																		
Slop			0.28ˆ*			0.31ˆ*			0.28ˆ*			0.27ˆ*			0.35ˆ**			0.29ˆ*
Curvature			-0.06			0.00			-0.07			-0.12			-0.02			0.01
Incongruence (M = -F) line																		
Slop			0.08			0.25			0.15			0.02			0.10			0.00
Curvature			0.06			0.19ˆ*			0.11			0.17ˆ*			0.13			0.09

^a^*n = 260. Unstandardized regression coefficients are reported. M, Model. ^∗^*p* < 0.05, ^∗∗^*p* < 0.01, ^∗∗∗^*p* < 0.001. Two-tailed tests*.

Hypothesis 1 suggests a linear relationship of one’s openness to relationship quality. In Model 2 (M2), we examined the linear relationship between self and partner openness to relationship quality. As can be seen in **Table 2**, male openness significantly predicted the relationship quality in the aspects of love, commitment, and intimacy, while the female openness showed no notable effect. In Model 3 (M3), we made the quadratic polynomial regression to show the curvature relationship and interaction between partners. Male openness holds a negative quadratic prediction in satisfaction and trust, and female openness holds a positive quadratic prediction in satisfaction, love, commitment, intimacy, and passion. Moreover, male and female openness interact in trust. Thus, Hypothesis 1 is partly supported.

**FIGURE 1:**
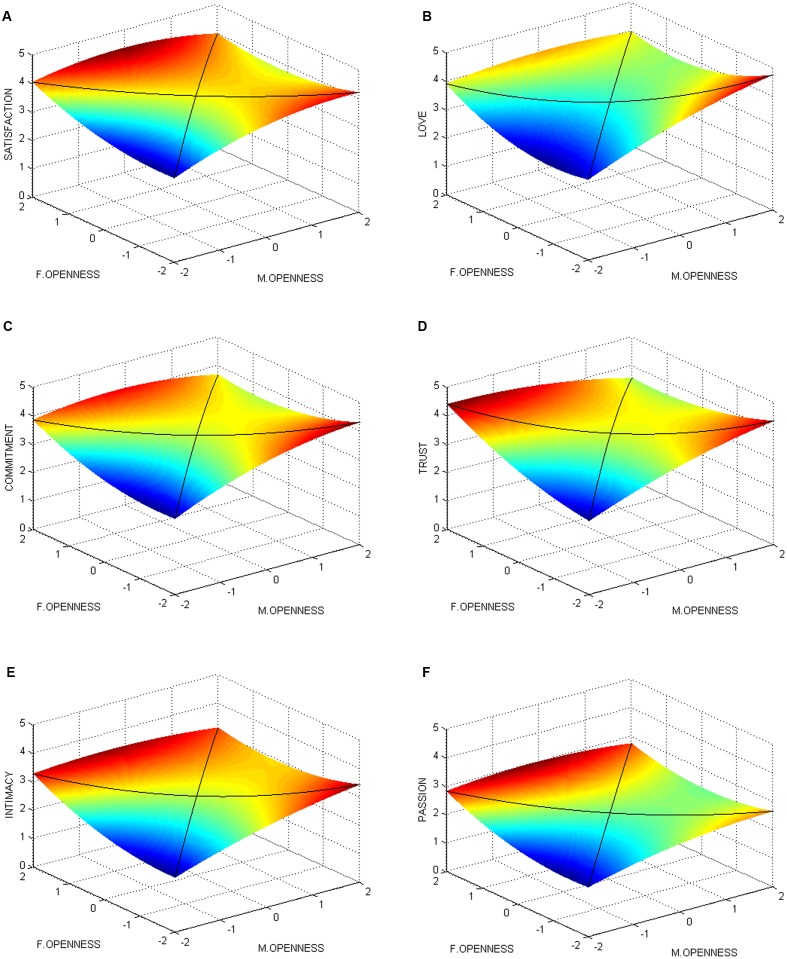
**Congruence effect and asymmetrical incongruence effect of males’ (M.OPENNESS) and females’ (F.OPENNESS) openness on relationship quality. (A)** Satisfaction; **(B)** love; **(C)** commitment; **(D)** trust; **(E)** intimacy; **(F)** passion.

Through analysis, although the response surface analyses needed a significant Δ*R*^2^ in Model 3 (M3), we conducted the analyses for all of the outcome variables. In addition, to facilitate interpretation of the results, a graphical response surface representation of the relationships between male openness, female openness, and relationship quality are provided based on the coefficients on six factors of relationship quality. The interactions of dyadic openness and each aspect of relationship quality are illustrated in **Figures 1A–F**. The crossed black solid lines on the surface represent how the degree of congruence (*X* = *Y*) and incongruence (*X* = -*Y*) between centered males’ openness (M.OPENNESS) and females’ openness (F.OPENNESS) relates to their relationship quality.

When partners reached congruence on openness (Male = Female, Hypothesis 3), relationship quality increased with a significant linear line (satisfaction = 0.28, *p* < 0.05; love = 0.31, *p* < 0.05; commitment = 0.28, *p* < 0.05; trust = 0.27, *p* < 0.05; intimacy = 0.35, *p* < 0.01; passion = 0.29, *p* < 0.05). Moving along the congruence line from the nearest to farthest corner, the lowest level of relationship quality was at the nearest corner, where couples were both low in openness, and increasingly higher toward the farthest corner, where couples were both high in openness. This means that couples similarly high in openness will have better relationship quality than couples similarly low in openness, thus supporting Hypothesis 3.

When openness was incongruent (Male = -Female, Hypothesis 4), the relationship quality curved upward with a significant u-shaped line (love = 0.19, *p* < 0.05; trust = 0.17, *p* < 0.05). The incongruence line extends from the left to right corner of the graph. Moving away from the center of the line, the difference between couples’ openness increased, and the relationship quality rose similarly to the left corner, where high females’ openness was combined with low males’ openness, and to the right corner, where low females’ openness was combined with high males’ openness. This indicated that when partners hold differential openness, they can experience better relationship quality than those with a moderate level of openness. Therefore, Hypothesis 4 is partially supported.

Hypothesis 2 suggested a general higher level of relationship quality in openness congruence dyads than openness incongruence ones. For congruence lines, results show a positive linear relationship, which means that low openness congruence dyads will have a lower relationship than the moderate dyads (Male = Female = 0). However, for incongruence lines, u-shaped and non-significant relationships exist. That is, the relationship quality of all of the incongruence dyads will be higher than or the same as that of the moderate incongruence dyads (Male = -Female = 0). Thus, these results could not demonstrate that the openness congruence is better than incongruence for relationships; we could also find the evidence from **Figures 1A–F**. Therefore, Hypothesis 2 is not supported.

## Discussion

This exploratory study illustrated openness and its fit effect on the close relationship quality of emerging adults. Using the quadratic polynomial regression and response surface analysis, our results are as follows.

First of all, considering the main effect of openness at the individual level, openness of men is more contributive to relationship quality, which is in line with previous studies (Holland and Roisman, 2008). However, female openness holds a positive quadratic effect. Women with high or low levels of openness could make their relationship quality higher than women with moderate openness. It is understandable that a female college student with a high openness trait may benefit the relationship quality. The interesting question is why a dyad with an open female partner could also be expected to have good relationship quality. We might find some corroborations from Chinese gender philosophy. A romantic relationship is influenced by hierarchical patriarchal norms in Chinese culture (Cheung and Halpern, 2010), and for a long time, “a woman without talent is virtuous” was widely accepted by not only ancient Chinese society (Shen and D’Ambrosio, 2016) but also contemporary Chinese college students (Rosen, 1992). A conservative woman may ease her partner’s perception of threat toward both her and the relationship.

The results showed that when a couple’s openness is congruent, it is better to be relatively high than relatively low. Relationship quality in the similar openness dyads is a rising line, suggesting that couples with relatively low openness would suffer more in the relationship. A high level of openness could raise partner’s relationship perception (Neyer and Voigt, 2004; Watson et al., 2004; Barelds, 2005), and couples high in openness could reciprocate and attain a satisfactory relationship. For couples low in openness, despite the fact that they would obtain stability in their lives and experiences, intolerance of ambiguity would increase (McCrae, 1996), and bidirectional inflexibility is more likely to block the interaction and construction of a better life. In conclusion, it can be easily foreseen that conflicts arise in relationships when individuals refuse to share their feelings (Lutz-Zois et al., 2006).

When dyadic openness is dissimilar, relationship quality in love and trust is a u-shaped line. The results illustrate that couples with complementary openness experience higher levels of love and trust than moderate openness couples. As mentioned earlier, self-openness could considerably raise the relationship satisfaction experienced by the partners (Neyer and Voigt, 2004). Considering that open individuals could create an inspiring and emotional relationship atmosphere, they could accelerate the establishment of trust. In addition, closed individuals will feel most satisfied with people who are open to their experiences because closed people lack a strong sense of self and could be simply destabilized by others (Gurtman, 1995). Hence, complementary openness mode could neutralize both sides and result in a higher level of relationship.

## Implication

For a long time, openness has traditionally been viewed as an intrapsychic trait, pertaining to individual differences in the structure and functioning of the mind but of little importance to social relationships (Mccrae and Costa, 1994; McCrae, 1996). Actually, the meta-analytic review also showed that the average correlation is about 0.03, which is the lowest among the big five factors (Malouff et al., 2010) and is consistent with the small importance in a high-quality intimate relationship. This exploratory research highlighted the role that openness plays in forming relationship quality and uncovered a more explicit personality mechanism shaping intimate relationships. Our study demonstrated that the effects of openness in an interpersonal relationship and intimate relationship might have been overlooked. The non-significant results may result from taking similarity or dissimilarity as a same score, which failed to distinguish various matching situations. We provided evidence that the similarity hypothesis and the complementary hypothesis were not contradictory to each other in the relationship between dyadic openness and relationship quality among emerging adults. Therefore, the results enrich our knowledge about openness and intimate relationship quality and also open a new scope when considering that personality matching should avoid using difference scores and take advantage of polynomial regression (Edwards, 2001). Furthermore, the interesting curvilinear relation of openness and relationship quality among Chinese emerging female adults sheds light on the culturally relevant gender ideology. However, we should note that this current research, which highlighted the joint effect of each dyad’s openness, is just an exploratory one. We hope that our study could inspire further research that focuses on the interpersonal aspect of openness.

Practically, during the developmental transition from adolescence to adulthood, one of the key missions of emerging adults is to develop an intimate relationship and prepare for marriage (Arnett, 2014). At the same time, the ability to have high-quality intimate relationships is a keystone of adult mental health and well-being (Reis et al., 2000; Noller et al., 2001). Hence, how to promote intimate relationship quality is a core issue that all emerging adults may be faced with. The research on assortative mating illustrated that individuals preferred partners who were similar to themselves in terms of personality characteristics, and this preference was especially strong for partners who were similar in openness and conscientiousness (Botwin et al., 1997). Our study reminds Chinese emerging adults that when selecting a mate, one should take his or her own personality, the potential partner’s personality, and his or her degree of matching into consideration. With openness as an example, our research illustrated that assortative mating would not always be the best choice. For those lower in openness, both male and female, it might be better to select an open mate to enjoy better dating relationship quality.

## Limitation and Further Study

Despite the implications of this exploratory study, there were still some limitations. Firstly, our results highlight the role of openness and its fit in high-quality relationships among couples who are in the stage of emerging adulthood. However, we still do not know whether the role of openness could be of the same importance in other life stages because the college environment encourages the students to be more open and highlights the role of openness (Robins et al., 2001). Thus, future studies must be expanded to include other life stages in order to test whether the vital role of openness fit still exists among couples in different life stages. Secondly, openness was originally considered to be an intelligence factor (Costa and McCrae, 1992; Baer et al., 2008) and was associated with academic performance (Vedel, 2014). Although the effect sizes of the relationship between openness and academic performance are small (Vedel, 2014; Kholin et al., 2016), because both academic performance and romantic relationships are of equal importance during emerging adulthood, and because involvement in a romantic relationship may result in decreasing academic performance (Giordano et al., 2008; Schmidt and Lockwood, 2015), it would be better to control academic performance in future studies. The third limitation of this research lies in the cross-sectional design. An increase in openness could be seen in the emerging adults throughout life in the university (Robins et al., 2001). Previous longitudinal research has demonstrated the personality effects on relationship, and vice versa, among adolescents and emerging adults (Neyer and Asendorpf, 2001; Robins et al., 2002; Asendorpf and Van Aken, 2003; Mund and Neyer, 2014; Schaffhuser et al., 2014b). From the cross-sectional design, we do not know to what extent the openness personality fit of two dyads predicts change in intimate relationships and how relationship experiences predict change in emerging adults’ openness trait. Future research should focus on the dyadic openness effect from a longitudinal perspective to explore the openness fit–relationship transaction. Furthermore, although our main intent here was not to examine the specific significant facet-level predictors of relationship quality, openness effect should be explored deeper in terms of its facets due to the fact that different facets of openness may have different effects on relationship quality (Noftle and Shaver, 2006). Finally, considering the robustness of the result, it would be better to control and compare the other four dimensions of big-five personality. In addition, the time spent together was treated as discrete in this study. In order to describe the variability of the investment in spousal interaction more effectively, future study should regard it as a continuous variable.

## Ethics Statement

This study was carried out in accordance with the recommendations of Ethics Committee of Institute of Psychology, Chinese Academy of Sciences with written informed consent from all subjects. All subjects gave written informed consent in accordance with the Declaration of Helsinki. The protocol was approved by the Ethics Committee of Institute of Psychology, Chinese Academy of Sciences.

## Author Contributions

YZ conducted the analyses, interpreted the data, and drafted the manuscript. KW participated in the study’s conception, design, and coordination and performed the measurement. SC participated in the study’s coordination and performed the measurement. JZ helped in the design of the study and helped to draft the manuscript. MZ conceived of the study, and participated in its design and coordination and helped to draft the manuscript. All authors read and approved the final manuscript. All authors read and approved the final version of this manuscript.

## Conflict of Interest Statement

The authors declare that the research was conducted in the absence of any commercial or financial relationships that could be construed as a potential conflict of interest.
